# A comparative study of cost and quality of life among Skeletal Related Events (SRE) and non-SRE among breast and prostate cancer patients in Malaysia

**DOI:** 10.1186/1472-6963-12-S1-P4

**Published:** 2012-11-21

**Authors:** Noraziani Khamis, Sharifa Ezat WP, Aljunid Syed, Zafar Ahmed

**Affiliations:** 1 National University of Malaysia, Cheras, Kuala Lumpur, Malaysia; 2 United Nations University International Institute for Global Health, Kuala Lumpur, Malaysia

## Background

The human skeleton is the most common organ to be affected by metastatic cancer (skeletal or bone metastases). The prevalence of skeletal disease is greatest in breast and prostate carcinoma. Bone metastases lesions weaken bone structure, causing a range of symptoms and complications. Sufferers of breast and prostate cancers may develop Skeletal related events (SRE). These SRE are defined as pain that require palliative radiotherapy or surgery to bone, hypercalcaemia, pathologic fractures, spinal cord compression and bone marrow failure. These complications contribute to a decline in patients' HRQOL (health related quality of life). However, information on treatment costs of breast and prostate cancers and multidimensional assessments of QOL are limited. Thus this study aim to obtain both cost and QOL of breast and prostate cancers patients and to determine their relationship with the patients sociodemograhic profiles (age, ethnicity, income) and disease profile (tumour types, cancer stages and SRE status).

## Objective

To determine the health care cost and QOL of breast and prostate cancer patients in Malaysia. The association of risk factors (sociodemographic and diseases profiles including SRE status) and cost and QOL will later be determined through statistical analysis.

## Methodology

This will be a societal approach, cost evaluation technique through a cross sectional study for a projected period of 9 months. Both qualitative and quantitative approach to estimate the health care cost of provider and patient’s cost through microcosting questionnaire method. Patient’s QOL will be using the locally validated EORTC-QLC-C30 questionnaire. Selected patients from oncologic and surgical wards, outpatients specialists clinics will be taken as respondents. Outcomes of study will include cost to treat a case of SRE and projected country's burden of SRE from both cancer. Other outcomes will be compared between SRE and non-SRE through measurements of cost per QALYs; cost per life years saved (LYS) and cost per deaths averted. Associated risk factors will be measured by bivariate and multivariate analyses with power of study of 80% and p value of 0.05.

## Expected result

The cost of treatment among prostate and breast cancer patient of lower disease compared to those of higher stages, positive bone metastasis and SRE positive. The QOL will also be expected to be higher among these groups. By similar disease stages for each cancer types, the QOL and costs are expected to be similar.

## Conclusion

This information will provide the cost burden of breast and prostate cancers for the country and will be used for strategic planning for the country. Patients with risk of lower QOL will also be identified for better risk assessment during cancer managements.

